# Propoxur enhances MMP-2 expression and the corresponding invasion of human breast cancer cells via the ERK/Nrf2 signaling pathway

**DOI:** 10.18632/oncotarget.19081

**Published:** 2017-07-07

**Authors:** Yunxiang Shi, Daizhi An, Yiping Liu, Qiong Feng, Xu Fang, Guilan Pan, Qiang Wang

**Affiliations:** ^1^ Center of Hygiene Assessment and Research, Institute of Disease Control and Prevention, Academy of Military Medical Sciences, Beijing 100071, China; ^2^ Beijing Municipal Public Security Hospital, Beijing Municipal Public Security Bureau, Beijing 100006, China; ^3^ Department of Physiology, BaoTou Medical College, Inner Mongolia University of Science and Technology, Baotou 014040, China

**Keywords:** propoxur, matrix metalloproteinase, nuclear factor E2-related factor 2, extracellular signal-regulated kinase 1/2

## Abstract

Propoxur is considered a prime etiological suspect of increasing tumor incidence, but the role is still undefined. In this study, two human breast cancer cells lines, MCF-7 and MDA-MB-231 cells, were used as cell models. Cells were respectively treated with 0, 0.01, 1, or 100 μM propoxur. PD98059, a MEK inhibitor, was administered to block the ERK/MAPK pathway. Migration and reactive oxygen species were measured by wound healing and Transwell assays, and flow cytometry. Protein expression and subcellular location were detected by western blotting and immunofluorescence staining, respectively. Results showed that propoxur treatment enhanced cell migration and invasion in a dose-dependent manner, while MMP-2 expression, but not MMP-9, was significantly increased in two cell lines. Meanwhile, the treatment increased intracellular reactive oxygen species, Nrf2 expression and nuclear translocation, and ERK1/2 phosphorylation. Inversely, inhibition of ERK1/2 activation with PD98059 significantly attenuated propoxur-induced Nrf2 expression and nuclear translocation. Moreover, PD98059 suppressed propoxur-induced cell migration and invasion, and MMP-2 overexpression. Collectively, these results indicate that propoxur can trigger reactive oxygen species overproduction, further promoting breast cancer cell migration and invasion by regulating the ERK/Nrf2 signaling pathways.

## INTRODUCTION

A large variety of synthetic organic chemicals of different chemical classes has been released into the environment over the last few decades [1–3]. Among them, carbamate insecticides, widely used in agricultural and non-agricultural fields, attract widespread concern due to their high biological activity, reduced rate of degradation, and long-term environmental persistence and high biomagnification in the food chain [4, 5]. Propoxur, a N-methylcarbamate ester (2-isopropoxyphenyl N-methylcarbamate), is a carbamate insecticide with a wide spectrum of applications. Propoxur is widely used in the control of cockroaches, flies, fleas, mosquitoes, bugs, ants, millipedes and other insect pests in food storage areas, fields, and houses. It is also used against migratory locusts and grasshoppers [6]. Therefore, it is very necessary for environmental researchers to spend more energy to study the adverse health effects arising from the application of propoxur.

Propoxur exposure can cause undesirable toxic effects in humans. The main toxic mechanism of propoxur is to inhibit the activity of acetylcholinesterase (AChE) [7]. The U. S. Environmental Protection Agency (EPA) has classified propoxur as moderately toxic (Toxicity Category II) for oral exposure and slightly toxic (Toxicity Category III) via dermal and inhalation routes of exposure. More importantly, it is also classified as a Group B2 probable human carcinogen by EPA [8]. Due to its estrogen-mimicking ability, propoxur is still considered a prime etiological suspect of increasing tumor incidence [5, 9], although a direct link has yet to be defined. Therefore, it is necessary to clarify the role of propoxur in the development of estrogen-related cancer, such as breast cancer, including cell proliferation, survival, invasion, metastasis, and angiogenesis.

Matrix metalloproteinases (MMPs), or matrixins, are a family of zinc endopeptidases [10]. One of the major implications of MMPs in cancer progression is their role in extracellular matrix (ECM) protein degradation, which allows cancer cells to migrate out of the primary tumor to metastasize [11]. Specifically, the activities of gelatinases MMP-2 and MMP-9 correlate with the invasive potential of cancer [12–14]. MMP-2 is involved in the activation of MMP-13 and the degradation of the basement membrane [15, 16]. MMP-9 is produced principally by osteoclasts and immune cells, which have been reported to be important for tumor growth [17, 18]. The expression levels of MMP-2 and MMP-9 are reported to be increased in bone metastasis nests of breast cancer [12]. In addition, reactive oxygen species (ROS) are transcriptional activators of ECM enzymes, such as urokinase plasminogen activator (uPA) and the MMP family of proteases [19, 20]. Furthermore, the expression of nuclear factor E2-related factor 2 (Nrf2), in addition to its antioxidative effects, was involved in the MMP activity in addition to cancer cell invasion and migration [21, 22].

Therefore, based on these data, we hypothesized that propoxur could reduce tumor cell migration and invasion through ROS-dependent secretion and activation of MMP-2 and MMP-9. To clarify the possible mechanism, we chose two breast cancer cell lines, MCF-7 and MDA-MB-231 cells, as cell models to test the effects of propoxur exposure on tumor cell migration and invasion. We also characterized the association between treatment and ROS activity, as well as the expression of MMP-2 and MMP-9. Furthermore, we explored the possible roles of the Nrf2 pathway in propoxur-induced cell migration and invasion *in vitro*.

## RESULTS

### Propoxur treatment enhances the migration and invasion potential of human breast cancer cells

**Figure 1:**
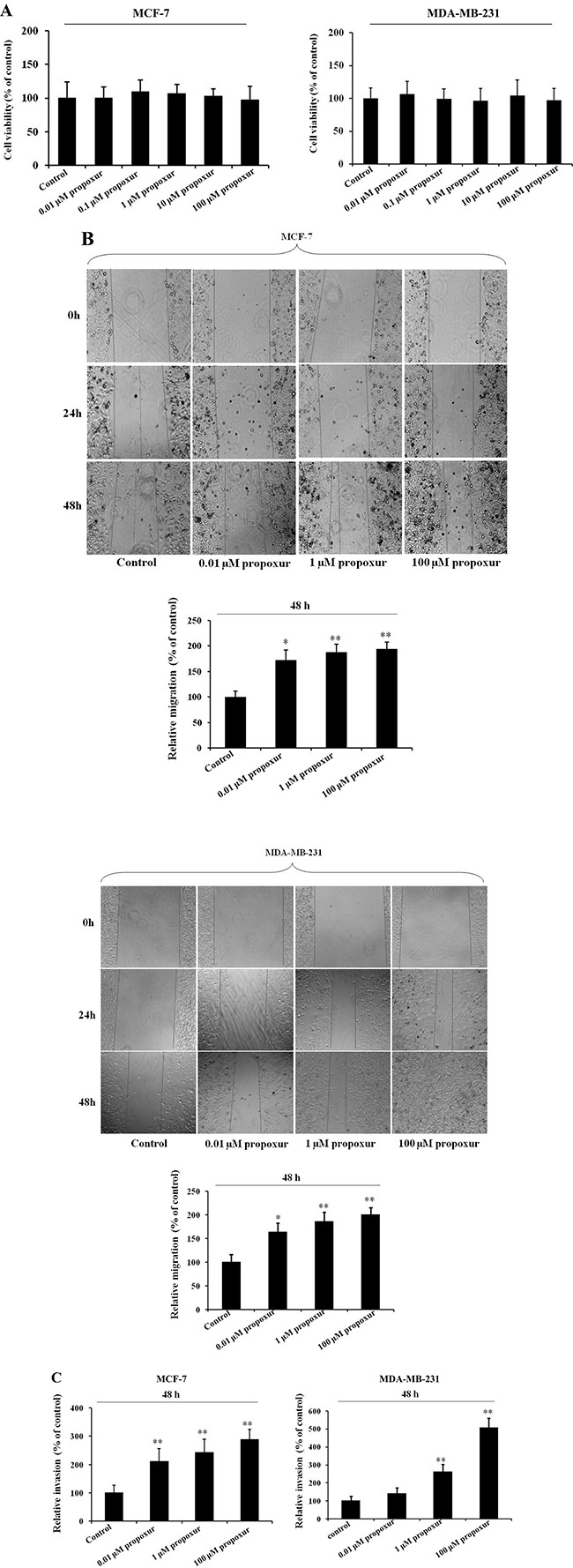
Propoxur treatment enhances the migration and invasion of human breast cancer cells Both MCF-7 and MDA-MB-231 cells were cultured without or with 0.01, 1 or 100 μM propoxur, and then the effect of propoxur on cell viability was determined by (**A**) MTT assays. (**B**) Wound healing migration assay of propoxur-treated and untreated breast cancer cells (magnification, ×100). Distances migrated by propoxur-treated and untreated cells were compared in three independent experiments. (**C**) Invasiveness of breast cancer cells (treated with or without propoxur) was quantified by Transwell assays.

Compared with untreated cells, the viability of MCF-7 and MDA-MB-231 cells was not significantly affected by 0.01, 1 or 100 μM propoxur treatment for 48 hours (Figure 1A). Morphological observation also indicated that 0.01, 1 and 100 μM propoxur exhibited no cytotoxicity in both MCF-7 and MDA-MB-231 cells (data not shown). Thus, the above concentrations were applied in the following experiments. Wound healing and Transwell assays were used to assess the effects of propoxur treatment on MCF-7 or MDA-MB-231 cell migration and invasion. As shown in Figure 1B, by wound healing assays, treatment with 0.01, 1 and 100 μM propoxur significantly enhanced cell migration in a dose-dependent manner. In Transwell assays, the results also showed a significant increase of cell invasion by treatment with propoxur in a dose-dependent manner. Compared with untreated cells, 0.01, 1 and 100 μM propoxur enhanced MCF-7 and MDA-MB-231 cell invasion by 110.8% and 41.0%, 142.4% and 162.1%, and 188.0% and 406.0%, respectively (Figure 1C). These data indicated that 0.01, 1 and 100 μM propoxur promoted cell migration and invasion, but had no significant cytotoxicity in MCF-7 and MDA-MB-231 cells.

### Propoxur treatment increases MMP-2 protein expression and intracellular ROS activity in human breast cancer cells

**Figure 2:**
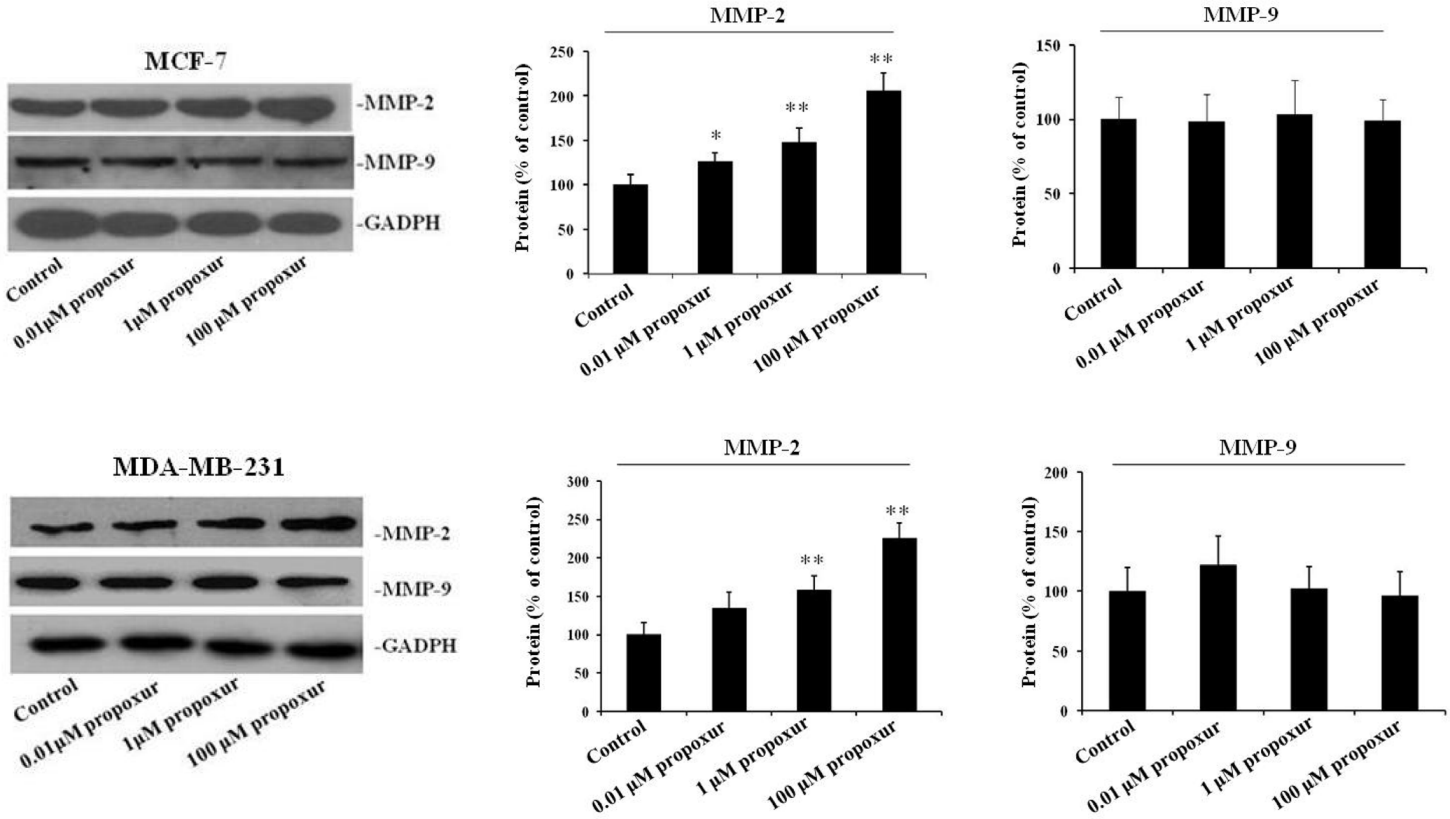
Propoxur treatment increases the expression levels of MMP-2 proteins in human breast cancer cells Both MCF-7 and MDA-MB-231 cells were cultured without or with 0.01, 1 or 100 μM propoxur. Western blot showed that propoxur treatment increased the levels of MMP-2 but not MMP-9. Representative blot and electrophoretogram of MMP-2 and MMP-9 proteins, respectively.

**Figure 3:**
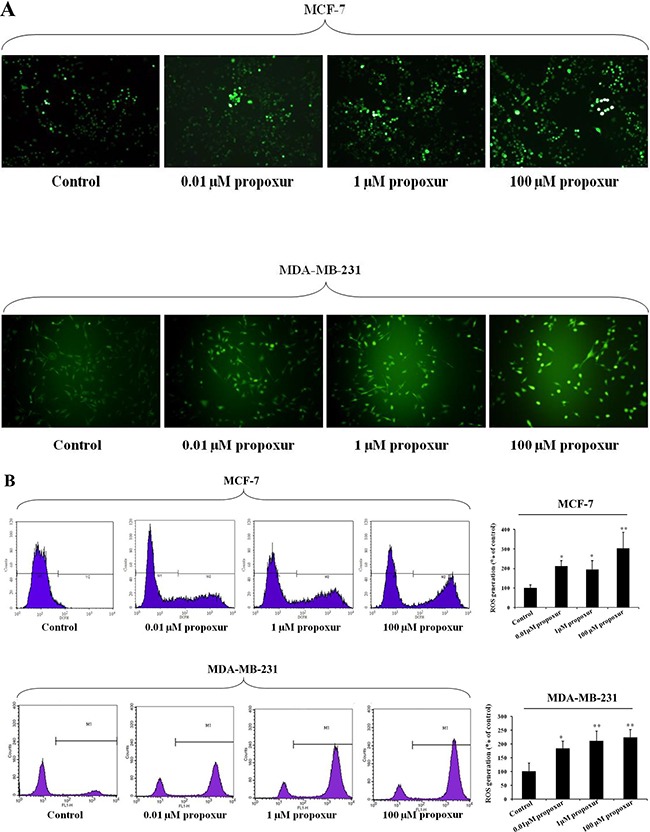
Propoxur treatment promotes intracellular ROS accumulation in human breast cancer cells Both MCF-7 and MDA-MB-231 cells were cultured without or with 0.01, 1 or 100 μM propoxur. Intracellular ROS was significantly increased by propoxur treatment. (**A**) Intracellular ROS was visualized under the confocal microscope with 100× magnification. (**B**) The mean fluorescence intensity of MCF-7 cells (with or without propoxur treatment) was quantified by flow cytometry.

MMP-2 and MMP-9 are thought to be important in tumor metastasis and tissue remodeling [11, 23]; therefore, the present study investigated the protein expression of MMP-2 and MMP-9 during the treatment of MCF-7 or MDA-MB-231 cells with propoxur. After cells were treated with 0.01, 1 and 100 μM propoxur for 48 hours, MMP-2 and MMP-9 protein expressions were detected by western blot analysis, respectively. The results showed there is a significant increase in the protein expression of MMP-2 in cells treated with 0.01, 1 or 100 μM propoxur for 48 hours (Figure 2). It is worthy to note that propoxur had no significant effect on the MMP-9 protein expression (Figure 2). Increased oxidative stress and ROS production have been associated with many human metastatic tumors [24], so rates of ROS production were also examined in the two breast cancer cells. By both immunofluorescence staining and flow cytometric analysis, the intracellular ROS levels showed a significant increase after treatment with 0.01, 1 and 100 μM propoxur (Figure 3). Thus, these results suggested that intracellular ROS production was implicated with propoxur-induced cell migration and invasion.

### Propoxur treatment augments Nrf2 expression and nuclear translocation and ERK1/2 phosphorylation

**Figure 4:**
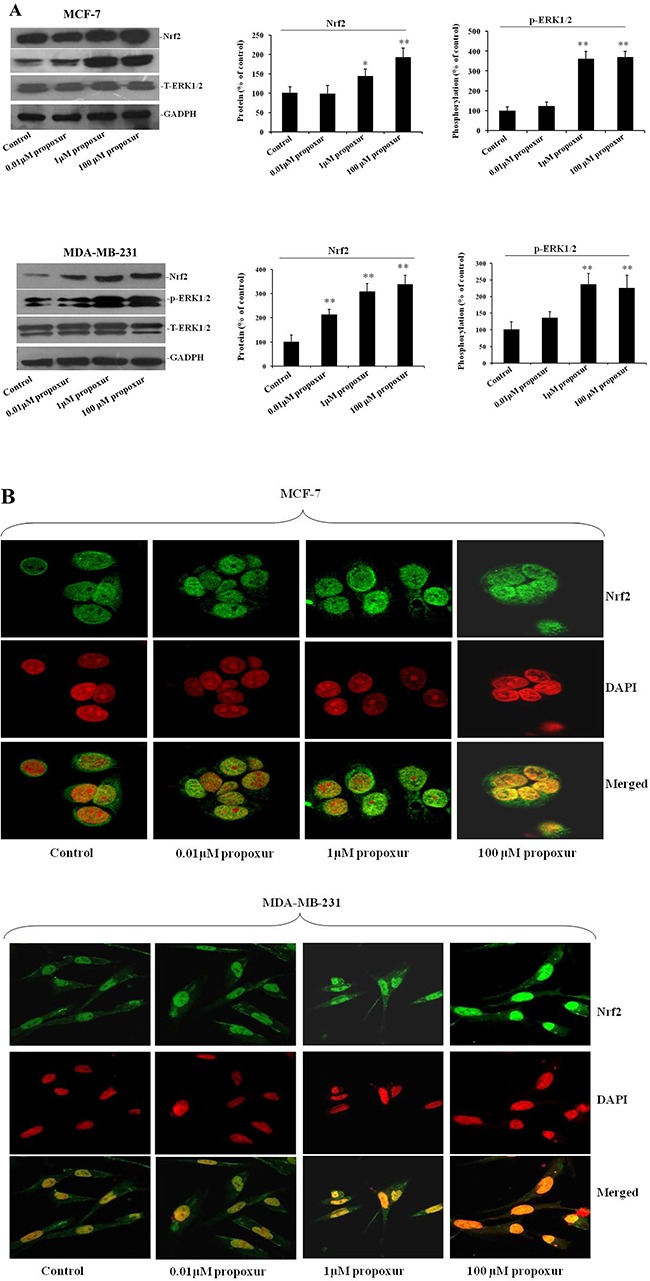
Propoxur augments Nrf2 expression and nuclear translocation and activates ERK1/2 activity Both MCF-7 and MDA-MB-231 cells were cultured without or with 0.01, 1 or 100 μM propoxur. (**A**) Representative blot and electrophoretogram of Nrf2, T-ERK1/2, and p-ERK1/2 proteins, respectively. (**B**) Representative confocal images of Nrf2 after different dosing of propoxur treatment (magnification, ×400). Following fixation, the cells were stained with a Nrf2 antibody (green) and DAPI (red).

Nrf2 is a primary cellular defense protein against the cytotoxic effects of oxidative stress [25], so we detected the Nrf2 protein expression during propoxur treatment in both MCF-7 and MDA-MB-231 cells. As shown by immunoblotting, levels of Nrf2 protein was significantly increased after 0.01, 1 and 100 μM propoxur treatment for 48 hours in a dose-dependent manner (Figure 4A). Moreover, Nrf2 upregulation was consistent with higher rates of ROS production. We also explored whether the activation of the ERK pathway, a downstream kinase of ROS, is involved with the propoxur-induced increase in ROS generation. By western blotting analysis, the result showed that the level of ERK1/2 phosphorylation significantly increased in propoxur-treated cells in a dose-dependent manner (Figure 4A). Nrf2, a redox-sensitive transcription factor, migrates into the nucleus and offers cytoprotection by inducing antioxidant enzymes [26]. Our results showed that the level of Nrf2 protein in the nucleus was gradually elevated with the dose of propoxur (Figure 4B). Control cells show sequestration of Nrf2 in the cytoplasm, revealing the unstressed state inside the cells. Cells treated with 0.01 and 1 μM propoxur showed partial nuclear localization of Nrf2, while extensive Nrf2 nuclear localization was observed in 100 μM propoxur treatment (Figure 4B).

### Inhibiting ERK1/2 activation by PD98059 suppresses propoxur-induced Nrf2 protein overexpression and nuclear translocation

**Figure 5:**
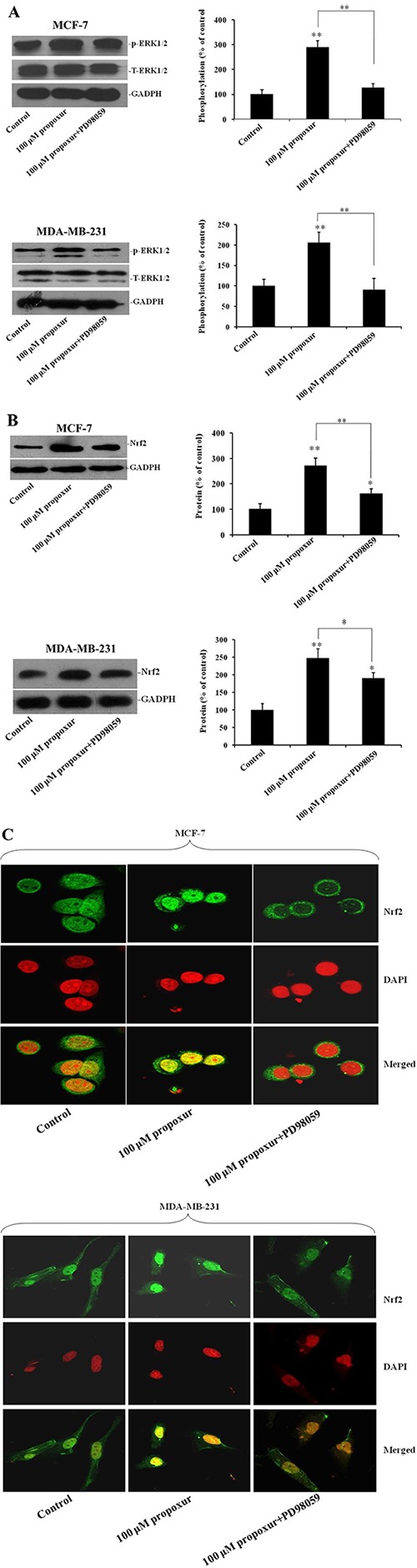
Inhibiting ERK1/2 activation by PD98059 suppresses the expression and translocation of Nrf2 protein Both MCF-7 and MDA-MB-231 cells were cultured without or with 100 μM propoxur, or 100 μM propoxur + 100 μM PD98059. (**A**) Representative blot and electrophoretogram of T-ERK1/2, p-ERK1/2 and Nrf2 proteins (**B**), respectively. (**C**) Representative confocal images of Nrf2 after different dosing of propoxur treatment (magnification, ×400). Following fixation, the cells were stained with a Nrf2 antibody (green) and DAPI (red).

PD98059 is a potent and selective inhibitor of MAP kinase kinases (MAPKK), MEK1 and MEK2 [27]. To further address the role of ERK1/2 pathways in propoxur-induced Nrf2 expression, 100 μM PD98059 was co-treated with 100 μM propoxur in both MCF-7 and MDA-MB-231 cells. By western blotting analysis, results showed that treatment with 100 μM PD98059 markedly blocked propoxur-induced ERK1/2 activation to near basal levels (Figure 5A). There were no changes in the levels of total ERK1/2 following treatment with 100 μM PD98059 (Figure 5A). In addition, compared with only propoxur-treated cells, PD98059 treatment also markedly attenuated propoxur-induced Nrf2 overexpression (Figure 5B). Furthermore, immunofluorescence staining assays showed that the propoxur-induced nuclear translocation of Nrf2 was also significantly attenuated by MEK1/2 inhibitor PD98059 (Figure 5C). These results suggest that propoxur-mediated Nrf2 induction is activated through ERK1/2 MAPK signal transduction pathways.

### Inhibiting ERK1/2 activation by PD98059 prevents propoxur-induced cell migration and invasion, and MMP-2 overexpression

**Figure 6:**
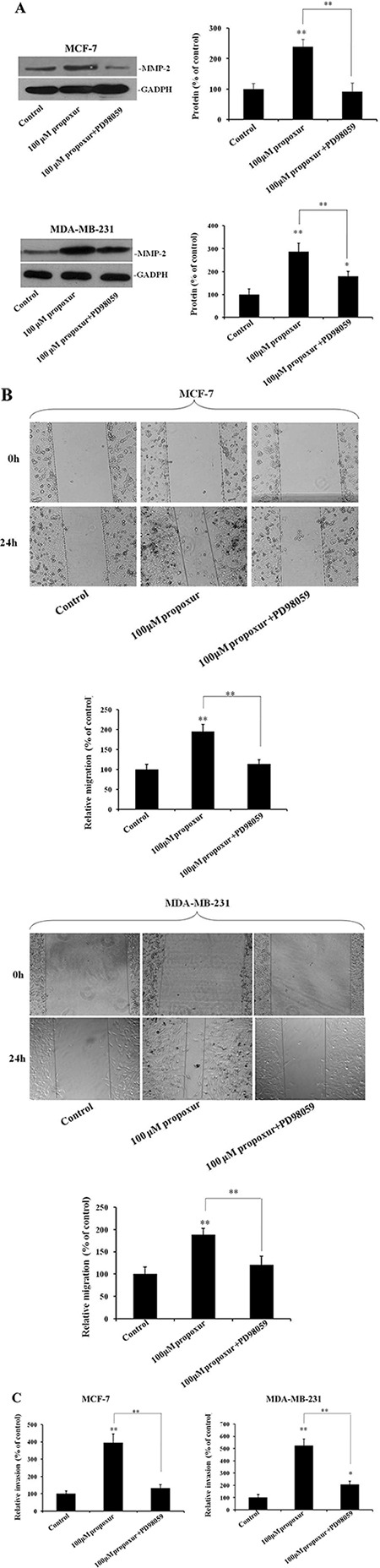
Inhibiting ERK1/2 activation by PD98059 prevents propoxur-induced MMP-2 overexpression, and cell migration and invasion Both MCF-7 and MDA-MB-231 cells were cultured without or with 100 μM propoxur, or 100 μM propoxur + 100 μM PD98059. (**A**) Representative blot and electrophoretogram of MMP-2 protein. (**B**) Wound healing migration assay of propoxur-treated and propoxur+PD98059 treated cells (magnification, ×100). Distances migrated by treated cells were compared in three independent experiments. (**C**) Invasiveness of cells was quantified by Transwell assays.

We also examined whether the ERK MAPK pathway plays a role in the regulation of propoxur-induced cell migration and invasion. Figure 6A showed that treatment of MCF-7 or MDA-MB-231 cells with 100 μM PD98059 significantly attenuated the increased MMP-2 protein induced by propoxur. Accordingly, by wound migration assays, treatment with 100 μM PD98059 resulted in a significant decrease of propoxur-induced migratory activity compared with that in only propoxur-treated cells (Figure 6B). Similarly, Transwell invasion assays showed a significant decrease of propoxur-induced invasiveness by treatment with 100 μM PD98059 compared with that in only propoxur-treated cells (Figure 6C). These results strongly indicate that the propoxur-induced cell migration and invasion could be activated by the ERK/Nrf2 signaling pathway.

## DISCUSSION

To the best of our knowledge, this is the first study to demonstrate that propoxur can enhance breast cancer migration and invasion *in vitro*, a tumor-promoting activity that may have important practical significance for the study of propoxur exposure on the health of the population.

At present, the carcinogenic and co-carcinogenic potential of propoxur is very limited and inconclusive. Although the mutagenic and genotoxic effects of propoxur are well established [28, 29], few studies have demonstrated the xenoestrogenic activity of propoxur in relation to estrogen-related cancer *in vitro*. Shukla et al. reported that propoxur has tumor-promoting activity but lacks complete carcinogenic and tumor-initiating activity in the mouse skin model of carcinogenesis [9]. Our results showed that a remarkable increase in cell migration and invasion was seen at lesser toxic concentrations of propoxur, signifying its capability to promote human breast cancer cell's metastasis. As we know, the initiation of metastasis involves the interaction of tumor cells with the ECM, through the process of cell-matrix adhesion and penetration out of the matrix [30]. MMP-9 and MMP-2 belong to gelatinase, which is one of five groups of the MMP family, based on the structure and substrate specificity [31]. In our study, propoxur had a significant effect on MMP-2 protein expression in a dose-dependent manner. Although MMP-9 has been demonstrated to play a central role in cancer metastasis, we found that the propoxur-induced MCF-7 cell migration was not mediated by MMP-9 protein.

In normal cells, there is a subtle balance between intracellular ROS and antioxidant capacity, which determines their destiny [32]. Yet cancer cells are characterized by elevated intracellular ROS stress, resulting from carcinogenesis stimulation, abnormal metabolic activity, and mitochondrial malfunction. The limited capacity of tumor cells to deal with the elevated ROS levels makes them vulnerable to oxidative stress [33]. Studies have demonstrated that ROS are transcriptional activators of ECM enzymes, such as uPA and MMP family of proteases [19, 20]. Furthermore, ROS can directly activate MMPs by oxidative modification of the cysteine residue [34, 35]. Our results showed that propoxur could trigger ROS overproduction, further promoting the expression of MMP-2 in both MCF-7 and MDA-MB-231 cells. Previous studies show that increased expression of phosphorylated ERK1/2 has been noted in various cancers, which can induce cancer cell proliferation and cancer progression [36]. ROS can activate downstream PI3K/AKT and ERK1/2 pathways that regulate HIF-1 and VEGF expression [37, 38]. Furthermore, ERK1/2 translocation to the nucleus to exert part of its biological activity is promoted by hypoxia [39]. In present study, the cell proliferation was not affected by propoxur treatment, but accompanied by increased expression of phospo-ERK1/2. Conversely, a decrease of ERK1/2 activity induced by a MEK1/2 inhibitor (PD98059) suppressed both ROS generation and MMP-2 protein expression. These results suggested that propoxur enhanced ROS-dependent migration and invasion of human breast cancer cells via upregulation of the ERK MAPK pathway.

Activation of antioxidant genes occurs via the Nrf2 signaling pathway under stress conditions to protect the cells/tissues from oxidative stress [40, 41]. Consistent with these reports, propoxur treatment markedly activated the Nrf2 signaling that increases Nrf2 protein level, and augments Nrf2 nuclear translocation, and enhances ROS generation, respectively. Under normal conditions, Nrf2 is sequestered in the cytoplasm by Kelch-like ECH-associated protein 1 (Keap-1). However, upon stimulation, Nrf2 translocates into the nucleus and recruits the small Maf (sMaf) protein. The Nrf2-sMaf heterodimer then binds to antioxidant response element (ARE), a cis-acting DNA regulatory element that activates the promoter region of many genes encoding phase II detoxification enzymes and antioxidants, such as heme oxygenase-1 (HO-1) and glutamate-cysteine ligase [42]. These enzymes exert antioxidant and cytoprotective property by eradicating the toxic free radicals/ROS in cells. In addition, activation of the Raf/ERK signaling cascade in human cancer cells has been demonstrated to be required for Nrf2 activation, which promotes Nrf2 nuclear translocation and binding to the specific DNA sequence [43, 44]. Our results clearly displayed that Nrf2 protein overexpression and nuclear translocation induced by propoxur were attenuated by inhibition of ERK phosphorylation by treatment with PD98059, which might lead to subsequently suppress propoxur-induced MMP-2 overexpression, and cell migration and invasion, respectively. Thus, our data confirmed that propoxur could promote breast cancer cell migration and invasion through intracellular ROS generation *in vitro*, which might be associated with the ERK/Nrf2 signaling pathway.

In summary, propoxur stimulates the breast cancer cell migration and invasion significantly. The cell migration and invasion activity of propoxur are associated with the increased expression of MMP-2 but not MMP-9. Notably, ROS is essential for propoxur-induced cell migration and invasion. It is very likely that ROS mediates MMP-2 expression through the ERK/Nrf2 signaling pathway. These findings may provide experimental evidence and explanation for carcinogenicity triggered by propoxur exposure. Furthermore, results hint that activity of MMP-2 induced by propoxur may be a critical regulator of propoxur-induced cell migration and invasion.

## MATERIALS AND METHODS

### Cell culture

Human breast cancer cell lines, MCF-7 and MDA-MB-231 cells, were obtained from the American Type Culture Collection (Manassas, VA, USA). The cells were cultured in Dulbecco's modified Eagle medium (DMEM) (Gibco, Carlsbad, CA, USA) supplemented with 10% fetal calf serum (FCS) (Hyclone, Waltham, MA, USA), 100 U/ml penicillin, and 100 μg/ml streptomycin, and incubated in a humidified atmosphere with 5% CO_2_ in air at 37°C.

### Cytotoxicity assay

MCF-7 or MDA-MB-231 cells (4×10^4^) were seeded in 96-well culture plates in quintuplicate and allowed to adhere overnight at 37°C. Then cells were incubated in medium (100 μL) containing 0.01, 1 or 100 μM propoxur. At the same time, cells were not exposed to propoxur as control group. After 48 hours of incubation, the growth medium was exchanged with 500 μg/ml methylthiazolyldiphenyl tetrazolium bromide (MTT), and the cells were incubated at 37°C for 4 hours. After removal of MTT, an aliquot of acidic isopropanol was added to the samples. The cell viability was analyzed by measuring the absorbance at 570 nm with DU 530 UV/Vis spectrophotometer (Beckman Coulter, Fullerton, CA, USA).

### Wound healing assay

Cells were seeded in 6-well plates and cultured to 80–90% confluence. The monolayer was then wounded by dragging a plastic pipette tip across the surface. Then, the cells were incubated with medium containing 0.01, 1 or 100 μM propoxur, or 100 μM propoxur +100 μM PD98059. The control cells were not exposed to propoxur. After 48 hours of incubation, phase contrast images of the wounds were recorded, and cells that had migrated into the wounded areas were counted to quantify the cell migration.

### Transwell invasion assay

Invasion assay was performed using Transwell chambers (24-well plates, 8-μm pore size, Corning, NY, USA). Cells at the density of 1 × 10^5^ cells per well were loaded into the insert containing medium with 0.01, 1 or 100 μM propoxur, or 100 μM propoxur + 100 μM PD98059. The lower chamber was added with a medium containing 10% FCS. After incubation for 48 hours at 37°C, the number of cells that migrated to the underside of the membrane was counted under a light microscope. Three independent experiments were carried out and each assay was performed in triplicate.

### ROS detection

Intracellular ROS was measured by flow cytometry using H_2_DCF-DA as a fluorescent probe. For flow cytometric measurements, cells were incubated with 10 μM H_2_DCF-DA for 30 minutes at 37°C, washed, resuspended in phosphate-buffered saline, and then analyzed by a FACSort (BD Biosciences, San Jose, CA, USA). The mean fluorescence intensity was quantified by CellQuest software (BD Biosciences, San Jose, CA, USA). We also imaged ROS with dihydroethidium using confocal microscopy. Confocal images were obtained with a Zeiss 710 LSM with an integrated META detection system.

### Immunofluorescence staining

After specific treatments, cells were washed with phosphate-buffered saline (PBS), then plated onto chamber slides and fixed with ice-cold 100% methanol for 20 min. The cell monolayer was pretreated with 0.3% (v/v) hydrogen peroxide in methanol for 30 min. The primary antibody of Nrf2 (Genetex, Irvine, CA, USA) was used at 1:200 of the working solution, and cells were incubated with the primary antibody solution for 1 hours at room temperature. The fluorescein isothiocyanate (FITC)-conjugated antibody (Santa Cruz Biotechnology, Santa Cruz, CA, USA) was used as a label for the immunofluorescence assay. After immunolabeling, cells were washed, stained with DAPI (Sigma-Aldrich, St Louis, MO, USA), and then viewed with fluorescent microscopy (Leica Microsystems, Wetzlar, Germany).

### Western blot analysis

After specific treatments, cells were homogenized in RIPA buffer containing a protease inhibitor cocktail (Roche, Basel, Switzerland). Cell lysates were determined for protein content using the Bradford method. Total proteins (15 μg) were separated by 12% sodium dodecyl sulfate-polyacrylamide gel electrophoresis and then electrotransferred by electroblotting onto nitrocellulose membranes. The blots were blocked using 5% blocking reagent in Tris-buffered saline with Tween-20 (TBS-T) for 1 hour at room temperature and then incubated with a primary antibody (1:2000) overnight at 4°C. After washing with TBS-T, the blots were incubated with a secondary antibody conjugated to horseradish peroxidase (1:2000) for 1 hour at 37°C. After extensive washing, the complexes were visualized using the West Pico chemiluminescent kit (Pierce, Rockford, IL, USA).

### Statistical analysis

Data are expressed as the mean ± standard deviation (SD). Differences between means were determined by one-way analysis of variance followed by a least significant difference test for multiple comparisons. A *p*-value of less than 0.05 was regarded as statistically significant (asterisk indicates *p* < 0.05; double asterisk indicates *p* < 0.01).
